# Comparison of the Quick Sequential Organ Failure Assessment (qSOFA) Score With the Glasgow-Blatchford Score (GBS) and Rockall Score (RS) in Predicting the Need for Intensive Care Among Acute Upper Gastrointestinal Bleeding Patients

**DOI:** 10.7759/cureus.80613

**Published:** 2025-03-15

**Authors:** Lissa Abraham, Susan Tharian, Ajmal Um

**Affiliations:** 1 Department of Emergency Medicine, Pushpagiri Institute of Medical Sciences and Research Centre (PIMSRC), Tiruvalla, IND

**Keywords:** acute upper gastrointestinal bleeding, glasgow-blatchford score, intensive care, rockall score, sequential organ failure assessment score

## Abstract

Objective: The present study aims to compare the quick sequential organ failure assessment (qSOFA) score, Glasgow-Blatchford score (GBS), and Rockall score (RS) in predicting intensive care needs among patients with upper gastrointestinal bleeding at a tertiary care medical college hospital in South India, thus helping to determine the most useful scores in predicting in-hospital adverse events among the three.

Methodology: This single-center, cross-sectional study was conducted from March 1, 2023, to February 29, 2024, after obtaining institutional review board approval from Pushpagiri Institute of Medical Sciences and Research Centre, Tiruvalla, Kerala, India. The investigator assessed the patients' characteristics (age, sex, presenting complaints, comorbidities), hemodynamic status, and laboratory variables at presentation in order to calculate the RS, GBS, and qSOFA for each patient. The patient's course in the hospital, including pre- and post-endoscopy characteristics and events such as recurrent bleeding, hematemesis, melena, mortality, intensive care, blood product transfusions, prolonged hospitalization, and the presence of hemodynamic instability, was documented.

Results: Out of the total 95 patients, 67.4% were aged over 60 years, with 66% being male. Chronic liver disease, hypertension, and diabetes mellitus were present in more than half of the patients. Hematemesis was the predominant presentation (51.6%), while tachycardia, tachypnea, and elevated blood urea nitrogen levels were noted more frequently. The patients were classified into low-risk and high-risk groups based on the three scores and were compared for intensive care unit (ICU) admissions, rebleeding, and death. A significant difference was noted between the GBS and the incidence of rebleeding (p-value: 0.043). The scores were compared for ICU admission using different parameters, and it was found that qSOFA had 100% sensitivity but very low specificity (10.59%), GBS had 92.11% sensitivity, with the lowest negative predictive value (NPV) at 33.33% and the highest positive predictive value (PPV) at 81.4%, and RS had a sensitivity of 96.08%, with intermediate NPV and PPV.

Conclusion: GBS provided the best overall prediction accuracy for the need for ICU admission in acute upper gastrointestinal bleeding patients, while qSOFA demonstrated extremely high sensitivity as a screening tool.

## Introduction

The term upper gastrointestinal bleeding (UGIB) refers to bleeding that occurs from the mouth to the duodenum, just proximal to the Treitz ligament. Peptic ulcer disease, esophageal ulcers, erosions of the upper gastrointestinal tract, variceal bleeding, gastroesophageal reflux disease, Mallory-Weiss tears, vascular lesions, and malignancies are the common causes of UGIB [1]. UGIB continues to be an important reason for emergency endoscopy, and the mortality remains significant, with rates of up to 11% at 30 days in patients with UGIB [2].

Identifying low- and high-risk patients through the use of a risk stratification tool is essential to the effective management of UGIB. Risk stratification can be helpful when it comes to initial triage, scheduling endoscopic procedures, and making admission/discharge decisions [3]. When there is UGIB, various risk scores are frequently used to predict unfavorable patient outcomes.

The Glasgow-Blatchford score (GBS) and Rockall score (RS) are the two most frequently utilized [4]. The GBS predicts whether patients with UGIB require immediate care [5]. The score is determined by considering the patient's admission blood urea, pulse, systolic blood pressure, and hemoglobin levels, as well as symptoms like syncope or melena and signs of cardiac failure or liver disease [6]. Rockall et al. [7] found that the following factors are independent predictors of mortality after acute upper gastrointestinal hemorrhage: age, shock, comorbidity, diagnosis, major stigmata of recent hemorrhage, and rebleeding. Thus, the RS was developed for stratifying acute UGIB [8].

In addition, patients with a high risk of mortality from sepsis can be more easily identified with the use of the quick sequential organ failure assessment (qSOFA) score. This score is an adaptation of the sequential organ failure assessment (SOFA) score, which is related to sepsis. The Glasgow Coma Scale (GCS), blood pressure, and respiratory rate are used by qSOFA to determine the severity of critical illness [9]. qSOFA is a quick and easy bedside scoring method. Its initial purpose was to predict sepsis-related mortality. When it comes to non-variceal UGIB, it can also be a useful prognostic indicator of serious clinical outcomes [10].

In patients with acute UGIB, the qSOFA score can predict in-hospital adverse events, thereby reducing hospital stay duration, cost, and time [11]. Hence, it is important to assess whether the qSOFA score is as effective as the other commonly used scores, such as GBS and RS, in predicting the need for intensive care among patients presenting with UGIB in the emergency department.

In a developing country like India, there is a critical need for a scoring tool that can be easily implemented to identify patients who require intensive care promptly. This would significantly improve patient survival, reduce complications, and lower hospital stay duration and associated costs. While existing risk stratification tools, such as the GBS and RS, have demonstrated certain levels of sensitivity and specificity in predicting outcomes in patients with UGIB, challenges remain in their broad application. For instance, the GBS and RS may be less practical in low-resource settings due to their complexity or limited adaptability to diverse patient populations. The qSOFA, on the other hand, offers a more straightforward and quicker assessment, but its effectiveness in predicting intensive care needs in UGIB patients remains uncertain. The present study, therefore, aims to compare qSOFA with GBS and RS in predicting intensive care requirements and in-hospital adverse events at a tertiary care hospital in South India. The study seeks to identify the most effective and feasible risk stratification method for this patient group by evaluating these tools in a real-world context.

## Materials and methods

Study design

This single-center, prospective longitudinal study was conducted at Pushpagiri Institute of Medical Sciences and Research Centre in Tiruvalla, Kerala, India. The research was carried out from March 1, 2023, to February 29, 2024, after obtaining institutional review board approval from the institute's Institutional Ethics Committee (approval number: PIMS&RC/E2/EM/211(3)/’2021). The study was conducted by the ethical principles of medical research outlined in the Declaration of Helsinki.

Sample size selection

This study's required minimum sample size was calculated assuming a 95% confidence interval and a 5% relative precision. This calculation was based on the mean and standard deviation of scores from a previous study by Taslidere et al. [12]. The formula used for calculating the sample size was N=(Zα/2)2pq/d2 where N represents the sample size, Z is the standard normal value at the desired level of confidence (usually at the 95% confidence level), p is the expected prevalence (0.5), q is 1-p, and d is the desired degree of precision (0.1).

Inclusion and exclusion criteria

This study included adult patients aged 18 years and older who presented with acute UGIB. Informed consent was obtained from all study participants before their enrollment. Patients who met the criteria for acute UGIB and had a confirmed diagnosis after clinical evaluation and endoscopy were eligible for inclusion.

The exclusion criteria were established to ensure a focused and homogeneous study population. Patients under 18 years were excluded due to the distinct physiological characteristics and different management approaches in pediatric populations. Additionally, patients who did not complete the necessary diagnostic evaluation or failed to undergo endoscopy were excluded, as this would limit the accuracy and completeness of the data. Pregnant women were excluded due to the potential risks associated with diagnostic procedures, such as radiation exposure and the need for specialized care.

Patients with human immunodeficiency virus (HIV) infection were excluded because the presence of comorbidities, such as liver disease or opportunistic infections, could interfere with the study's outcomes. Similarly, patients who had experienced trauma were excluded, as traumatic injuries could confound the study results, especially when it comes to managing bleeding and assessing risk factors for UGIB.

Finally, patients who were terminally ill, with conditions that would result in a life expectancy of less than six months, or who were unlikely to benefit from the study due to severe comorbidities were also excluded. This ensured that the study focused on patients with a reasonable prognosis who could provide reliable data on the outcomes of UGIB management.

Operational definitions for this study

UGIB

UGIB was defined as blood loss originating from a gastrointestinal source above the Treitz ligament. This bleeding was typically evidenced by hematemesis (blood-stained vomit) or melena (dark, tarry stools). In addition to these primary symptoms, patients may have exhibited signs of blood loss, including weakness, exhaustion, and syncopal episodes. These clinical presentations were used to uniformly identify UGIB across all patients, ensuring consistency in diagnosis and classification. For this study, the identification of UGIB was based solely on these clinically relevant symptoms and confirmed by endoscopic evaluation [13].

Common etiologies of UGIB: The common etiologies of UGIB included peptic ulcer disease, esophagitis, gastritis, duodenitis, varices, portal hypertensive gastropathy (PHG), angiodysplasia, Dieulafoy lesion, gastric antral vascular ectasia, Mallory-Weiss tears, post-surgical bleeds, and upper gastrointestinal tumors. These underlying causes were identified based on clinical presentation, endoscopic findings, and relevant patient history. A thorough understanding of these etiologies was necessary for patient classification and treatment strategy determination to provide the most appropriate clinical care [14].

Massive Hemorrhage

Massive hemorrhage was defined by the presence of hemodynamically significant manifestations, especially hypotension, occurring after substantial blood loss. This condition referred to a loss of 20-25% of the intravascular blood volume, resulting in clinical signs of shock. These signs typically included a systolic blood pressure of less than 90 mmHg or a sustained decrease in systolic blood pressure of more than 40 mmHg from baseline despite adequate fluid resuscitation. Additionally, tachycardia (heart rate greater than 100 beats per minute), altered mental status due to hypoperfusion of the brain, and reduced urine output caused by renal hypoperfusion were also considered indicative of massive hemorrhage. These parameters were rigorously monitored throughout the study to ensure the accurate classification of massive hemorrhage. If a patient exhibited any combination of these clinical signs, along with laboratory evidence of anemia (e.g., a significant drop in hemoglobin), they were considered as experiencing massive hemorrhage. The study ensured consistency by training clinicians to recognize and document these parameters, with any deviations from the standardized criteria carefully noted and justified [15].

GBS

The GBS was used to assess the need for medical intervention in patients presenting with acute UGIB. A GBS of 0 indicated the patient was at low risk and could be considered for safe early discharge. A GBS score above 0 signified the need for medical intervention, such as transfusion, endoscopy, or surgery. A GBS greater than 6 indicated the need for blood transfusion and urgent inpatient investigations. These cut-off values were established based on prior research and clinical guidelines, where a score of 0 demonstrated a low risk for adverse events and a score more excellent than 6 suggested significant clinical intervention was required [16].

RS

The RS was utilized pre- and post-endoscopy to assess the risk of adverse outcomes in UGIB patients. A pre-endoscopy RS of 3 or less predicted a low risk of complications, while a post-endoscopy RS of 8 or more suggested a high mortality risk. An RS greater than 3 was associated with poor outcomes, such as rebleeding, the need for surgery, or death. The cut-off values for RS were set based on well-established thresholds in the literature, with a pre-endoscopy score of 3 indicating low risk and a post-endoscopy score of 8 or higher indicating high mortality risk [17,18]. This scoring system was rigorously applied, with pre-endoscopy scores calculated upon initial presentation and post-endoscopy scores calculated after the patient underwent endoscopic evaluation.

SOFA

The SOFA score was used to evaluate organ failure in patients, particularly those with suspected sepsis. A SOFA score of 2 or higher was associated with a 10% risk of in-hospital mortality. This study's SOFA criteria were rigorously applied, assessing parameters such as respiratory rate, blood pressure, liver function, and renal function. All data were recorded during patient admission and at regular intervals throughout their hospital stay to track organ function and ensure consistent data collection across all participants [19].

qSOFA

The qSOFA score utilized simple bedside criteria to identify patients with suspected infections likely to experience poor outcomes. A qSOFA score of 0 indicated low risk, while a score of 3 had more substantial predictive power for rebleeding and other adverse events during hospitalization. The qSOFA score was assessed upon patient presentation and monitored throughout their hospital course to guide patient care and intervention decision-making. The cut-off for qSOFA was set at 3, as this threshold demonstrated a higher predictive value for poor outcomes, such as rebleeding or mortality, among patients with acute UGIB [20].

Intensive care unit (ICU) admission criteria

ICU admission was determined based on clinical judgment, with key factors including hemodynamic instability, such as persistent hypotension despite adequate fluid resuscitation. The need for intensive monitoring due to the severity of UGIB and the requirement for invasive procedures like endoscopic interventions or surgery were also considered. The criteria for ICU admission were based on specific clinical parameters, including ongoing hemodynamic instability or the requirement for continuous monitoring or therapeutic interventions. Patients with significant comorbidities, such as liver cirrhosis, renal failure, or cardiovascular instability, were also prioritized for ICU admission, as these conditions could complicate recovery and necessitate specialized care. These criteria were consistently applied to ensure that ICU admissions were appropriate and based on the clinical needs of the patients.

Data collection method

Eligible patients who met the inclusion criteria were enrolled in the study after providing informed consent. The investigator assessed various patient characteristics, including age, sex, presenting complaints, comorbidities, hemodynamic status, and laboratory variables at presentation. Laboratory variables included complete blood count (CBC), serum electrolytes, renal function tests (e.g., serum creatinine, blood urea nitrogen), liver function tests (e.g., aspartate aminotransferase (AST), alanine transaminase (ALT), bilirubin), coagulation profiles (e.g., prothrombin time (PT), activated partial thromboplastin time (aPTT), international normalized ratio (INR)), and arterial blood gas (ABG) values. These laboratory results, along with clinical assessments, were used to calculate the RS, GBS, and qSOFA for each participant.

The study also documented the patient's hospital stay, including pre- and post-endoscopy characteristics and events such as recurrent bleeding, hematemesis, melena, mortality, intensive care admission, blood product transfusions, prolonged hospitalization, and hemodynamic instability.

Follow-up of discharged patients

Patients who were discharged home were followed up for 30 days post-discharge to monitor for any adverse outcomes, including rebleeding, recurrence of symptoms, and any complications related to the initial presentation of acute UGIB. Follow-up was ensured through scheduled outpatient visits at seven, 14, and 30 days post-discharge. Additionally, hospital staff (study coordinators or research nurses) contacted patients via phone every seven days during the follow-up period to assess their health status and collect data on adverse events. Patients were contacted by phone to ensure comprehensive data collection in case of missed follow-up visits.

Statistical analysis

Data analysis was conducted using IBM SPSS Statistics for Windows, Version 25.0 (Released 2017; IBM Corp., Armonk, New York, United States). A statistical significance level was set at 5%. Continuous variables were summarized using mean and standard deviation, while categorical variables were summarized using frequency and percentage. A p-value of less than 0.05 was considered statistically significant for determining the relevance of the findings.

Additionally, sensitivity, positive predictive value (PPV), and negative predictive value (NPV) tests were performed to evaluate the performance of the scoring systems (GBS, RS, and qSOFA) in predicting adverse outcomes and ICU admission. These tests helped assess the ability of each scoring system to correctly identify true positives (sensitivity), correctly identify patients without adverse outcomes (NPV), and predict the likelihood of adverse events when a positive score was observed (PPV).

## Results

The study found that 49 (51.6%) patients were in the 60-79-year age group, followed by 31 (32.6%) patients younger than 60. A total of 15 (15.8%) patients were aged 80 years or older. Among the 95 patients, 63 (66%) were male, and the remaining 32 (34%) were female. The various comorbidities were enlisted, and their incidence was noted (Table 1).

**Table 1:** List of comorbidities found in the participants

Comorbidities	Total number of patients (N=95)
Heart failure	6 (6.32%)
Malignancy	7 (7.37%)
Chronic kidney disease	10 (10.53%)
Cerebrovascular accident	15 (15.79%)
Coronary artery disease	23 (24.21%)
Hypertension	49 (51.58%)
Chronic liver disease	52 (54.74%)
Diabetes mellitus	52 (54.74%)

Out of the 95 patients in the study, four patients were on anticoagulant medications, and 24 were on antiplatelet medications for various indications. Fifty-nine patients (62.1%) presented with hematemesis, while 49 (51.6%) had melena. Syncope was noted in 15 patients. ICU admissions were recorded for 86 (90.5%) out of the 95 patients (90.5%). Rebleeding occurred in 14 (14.7%) patients, and four patients died during treatment.

The study found that the mean heart rate of the study population was 95.91±19.08 beats per minute, with a minimum of 59/min and a maximum of 150/min. The mean respiratory rate was 21.49±3.4 per minute, with a minimum of 16/min and a maximum of 30/min. The study also noted that the systolic blood pressure ranged from 50 to 190 mmHg, with a mean of 118.79±29.659 mmHg. Blood urea nitrogen levels ranged from 1 to 248 mg/dL, with a mean of 56.46±42.965 mg/dL. Hemoglobin levels ranged from 4 to 20 g/dL, averaging 9.40±2.927 g/dL. Platelet counts ranged from 1 to 6 lakh per cubic millimeter, with a mean count of 1.95±0.985 lakh per cubic millimeter. In the study, 46 (48.4%) patients received blood transfusions. The length of hospital stay ranged from one to 25 days, with a mean duration of 6.40±3.581 days (Table 2).

**Table 2:** Clinical profile of patients

Parameters	Mean±SD	Range (minimum to maximum)
Heart rate (beats per minute)	95.91±19.08	59-150
Respiratory rate (per minute)	21.49±3.4	16-30
Systolic blood pressure (mmHg)	118.79±29.659	50-190
Blood urea nitrogen (mg/dL)	56.46±42.965	1-248
Hemoglobin levels (g/dL)	9.40±2.927	4-20
Platelet count (per cubic millimeter)	1.95±0.985	100000-600000
Length of hospital stay (days)	6.40±3.581	1-25

Based on the qSOFA score, 85 out of the 95 patients were classified as high risk, while the remaining 10 were at low risk for in-hospital adverse events. Among the 85 low-risk patients, 76 (89.4%) were admitted to the ICU, rebleeding occurred in 12 (14.1%) patients, and three died. Among the 10 high-risk patients, all were admitted to the ICU, two patients had rebleeding, and one died. Statistically significant differences were not observed in any of these findings (p-values: 0.279, 0.620, and 0.279, respectively). Based on the GBS, 19 patients were categorized as low risk, and 76 were categorized as high risk for interventions. Of the 19 low-risk patients, 16 (84.2%) were admitted to the ICU, none experienced rebleeding, and one patient died. Among the 76 high-risk patients, 70 (92.1%) were admitted to the ICU, rebleeding occurred in 14 patients, and three patients died. No significant differences were noted in the association between GBS and ICU admission (p-value: 0.293) or between GBS and death (p-value: 0.798). However, a statistically significant difference was observed in the association between GBS and the incidence of rebleeding (p-value: 0.043). Using the RS, four patients were classified as low risk, 40 as intermediate risk, and 51 as high risk. Of the four low-risk patients, three were admitted to the ICU (75%), with no cases of rebleeding or death. Among the 40 intermediate-risk patients, 34 were admitted to the ICU, rebleeding occurred in three, and two patients died. Of the 51 high-risk patients, 49 (96.1%) were admitted to the ICU, rebleeding occurred in 11, and two patients died. None of these findings were statistically significant (p-values: 0.112, 0.119, and 0.883) (Table 3).

**Table 3:** Association of clinical scores with ICU admission, rebleeding, and death ^a^: low risk; ^*^: significant p-value ICU: intensive care unit; qSOFA: quick sequential organ failure assessment

Parameters	ICU admission N (%)	P-value	Rebleeding N (%)	P-value	Death N (%)	P-value
qSOFA score^a^	76 (89.4%)	0.27	12 (14.1%)	0.62	3 (3.5%)	0.27
Glasgow-Blatchford score	70 (92.1%)	0.29	14 (18.4%)	0.79	3 (3.9%)	0.043^*^
Rockall score	49 (96.1%)	0.112	11 (21.5%)	0.119	2 (3.9%)	0.88

The qSOFA score demonstrated the highest sensitivity (100%) and NPV (100%) but had the lowest specificity (10.59%) and accuracy (20%). The GBS showed high sensitivity (92.11%) and the highest accuracy (76.84%), with a PPV of 81.40%. The RS exhibited a sensitivity of 96.08%, specificity of 15.91%, and accuracy of 58.95%. Among all scores, GBS appeared to have the best overall balance between sensitivity, PPV, and accuracy among the three scores (Table 4).

**Table 4:** Evaluation of predictive accuracy of the qSOFA, Glasgow-Blatchford score, and Rockall score qSOFA: quick sequential organ failure assessment

Evaluation parameter	qSOFA	Glasgow-Blatchford score	Rockall score
Sensitivity	100%	92.11%	96.08%
Specificity	10.59%	15.79%	15.91%
Positive predictive value	11.63%	81.40%	56.98%
Negative predictive value	100%	33.33%	77.78%
Accuracy	20%	76.84%	58.95%

## Discussion

The present study, therefore, aims to compare qSOFA with GBS and RS in predicting intensive care requirements and in-hospital adverse events at a tertiary care hospital in South India. The study seeks to identify the most effective and feasible risk stratification method for this patient group by evaluating these tools in a real-world context.

In the present study, the majority of patients (67.4%) were elderly (aged over 60 years), and they were predominantly male. Older individuals are more vulnerable to acute UGIB due to the higher prevalence of comorbidities and polypharmacy, as noted by Hearnshaw and colleagues [21]. There is considerable evidence suggesting a higher incidence of acute UGIB in males than females, likely due to differences in lifestyle [22]. Among the comorbidities, we found diabetes (55%), chronic liver disease (55%), and hypertension (51%) to be associated with more than half of the patients. Wang and colleagues described a higher risk of acute UGIB in patients with diabetes, which may be attributed to the insufficient microcirculation associated with diabetes mellitus [23]. Chronic liver disease, due to its complications, including portal hypertension and thrombocytopenia, is a common cause of acute UGIB. Hypertension has also been found to increase the risk of submucosal UGIB [24]. The use of antiplatelet and anticoagulant medications is known to increase the risk of gastrointestinal bleeding, and we observed similar trends in our study.

Based on available scientific literature, we found hematemesis to be the most common presenting symptom (62.1%) of acute UGIB [25]. Our study also found syncope, tachycardia, and tachypnea to be present in a substantial number of patients upon admission. These findings were attributed to hypovolemia and hypoperfusion following significant UGIB [26]. Higher mean blood urea nitrogen levels were noted in our study. This could be attributed to the increased amount of digested blood in the gastrointestinal tract being converted to protein products, which are later metabolized to blood urea nitrogen by the liver [27]. Our study's high blood transfusion rates (48.4%) aligned with the need to maintain hemoglobin levels between 7 and 9 g/dL to reduce mortality in acute UGIB [28].

When the three scores, namely, qSOFA, GBS, and RS, were compared separately for significant associations with outcomes (ICU admission, rebleeding, and in-hospital death), we found that only the GBS score exhibited a significant association with rebleeding. At the same time, the other findings were not significant.

In the final evaluation of parameters, the qSOFA score accurately identified all patients who required ICU admission with perfect sensitivity (100%) and NPV (100%). However, its low PPV (11.63%) and specificity (10.59%) indicated that it commonly provides false positives, predicting ICU admission for patients who do not require it. The overall accuracy of just 20% shows that, although qSOFA is effective at identifying patients who require ICU care, its imprecision renders it unreliable for making precise predictions about ICU admission.

With a sensitivity of 92.11%, the GBS accurately identified 92.11% of patients who required ICU admission. With a specificity of 15.79%, it accurately identified 15.79% of patients who did not require ICU admission. The GBS had a PPV of 81.40%, meaning 81.40% of patients predicted to require ICU admission did. The NPV of 33.33% suggests that 33.33% of patients expected not to require ICU admission did not. The overall accuracy of GBS was 76.84%, indicating its overall effectiveness in predicting ICU admissions.

The RS correctly identified 96.08% of patients who required ICU admission. With a specificity of 15.91%, it identified 15.91% of patients who did not require ICU admission. The RS had a PPV of 56.98%, indicating that 56.98% of patients predicted to require ICU admission did. With an NPV of 77.78%, it accurately predicted that 77.78% of patients expected not to require ICU admission did not. The overall accuracy of the RS in forecasting ICU admissions was 58.95%, meaning 58.95% of the predictions were correct. Our study's results are comparable to those of available studies, which demonstrate the superior accuracy of GBS and RS compared to qSOFA [29].

Limitations

The study design, being cross-sectional, could not assess the long-term outcomes and progression of the etiological causes of acute UGIB. Only 95 patients were included in the study, and the data collection was conducted at a single hospital. Therefore, our results' prognostic stratification and significance require further large-scale research for confirmation and generalizability. This study had no control group, which would have made the findings more robust.

## Conclusions

Our study found that acute UGIB was more common in elderly patients (above 60 years), males, and those with diabetes mellitus, chronic liver disease, or hypertension. Hematemesis was the most common presenting symptom, while tachycardia, tachypnea, and increased blood urea nitrogen levels were frequently observed. The GBS demonstrated the best overall prediction accuracy for ICU admission. At the same time, qSOFA proved to be an extremely sensitive screening tool for identifying acute UGIB patients who may require ICU admission.
